# Green Synthesis and Characterization of Ginger-Derived Silver Nanoparticles and Evaluation of Their Antioxidant, Antibacterial, and Anticancer Activities

**DOI:** 10.3390/plants13091255

**Published:** 2024-04-30

**Authors:** Shweta Mehrotra, Vinod Goyal, Christian O. Dimkpa, Vinod Chhokar

**Affiliations:** 1 Department of Bio and Nano Technology, Guru Jambheshwar University of Science & Technology, Hisar 125001, India; 2 Department of Botany and Plant Physiology, CCS Haryana Agricultural University, Hisar 125001, India; 3 Department of Analytical Chemistry, Connecticut Agricultural Experiment Station, New Haven, CT 06511, USA

**Keywords:** antibacterial activity, antioxidant activity, cytotoxicity, ginger, green synthesis, phytochemical properties, nanoparticles

## Abstract

The efficacy, targeting ability, and biocompatibility of plant-based nanoparticles can be exploited in fields such as agriculture and medicine. This study highlights the use of plant-based ginger nanoparticles as an effective and promising strategy against cancer and for the treatment and prevention of bacterial infections and related disorders. Ginger is a well-known spice with significant medicinal value due to its phytochemical constituents including gingerols, shogaols, zingerones, and paradols. The silver nanoparticles (AgNPs) derived from ginger extracts could be an important non-toxic and eco-friendly nanomaterial for widespread use in medicine. In this study, AgNPs were biosynthesized using an ethanolic extract of ginger rhizome and their phytochemical, antioxidant, antibacterial, and cytotoxic properties were evaluated. UV–visible spectral analysis confirmed the formation of spherical AgNPs. FTIR analysis revealed that the NPs were associated with various functional biomolecules that were associated with the NPs during stabilization. The particle size and SEM analyses revealed that the AgNPs were in the size range of 80–100 nm, with a polydispersity index (PDI) of 0.510, and a zeta potential of −17.1 mV. The purity and crystalline nature of the AgNPs were confirmed by X-ray diffraction analysis. The simple and repeatable phyto-fabrication method reported here may be used for scaling up for large-scale production of ginger-derived NPs. A phytochemical analysis of the ginger extract revealed the presence of alkaloids, glycosides, flavonoids, phenolics, tannins, saponins, and terpenoids, which can serve as active biocatalysts and natural stabilizers of metallic NPs. The ginger extracts at low concentrations demonstrated promising cytotoxicity against Vero cell lines with a 50% reduction in cell viability at 0.6–6 μg/mL. When evaluated for biological activity, the AgNPs exhibited significant antioxidant and antibacterial activity on several Gram-positive and Gram-negative bacterial species, including *Escherichia coli*, *Bacillus subtilis*, *Pseudomonas aeruginosa*, and *Staphylococcus aureus*. This suggests that the AgNPs may be used against multi-drug-resistant bacteria. Ginger-derived AgNPs have a considerable potential for use in the development of broad-spectrum antimicrobial and anticancer medications, and an optimistic perspective for their use in medicine and pharmaceutical industry.

## 1. Introduction

*Zingiber officinale*, commonly known as ginger and belonging to the family Zingiberaceae, is a plant rhizome widely used as a spice and a traditional medicine with minimal side effects to treat various types of pain, arthritis, indigestion, cold, cough, rheumatism, diabetes, obesity, and cancer. This medical property is due to the broad range of phytochemicals such as gingerols, shogaols, zingerone, paradol, terzingiberene, pineol, terpenes, geraniol, and limonene, among others [1]. In fact, a wide range of organic compounds and phytochemicals have been reported in ginger extract by gas chromatography-mass spectrometry [2]. The fresh rhizome of ginger contains the main active constituents known as gingerols. Shogaols are the primary pungent components found in dried ginger. Shogaols are derived from the drying process of gingerols [3]. 6-gingerol and its dehydrated form, 6-shogaol, are the major pharmacologically active components of ginger. Ginger contains numerous lipids; waxes; carbohydrates; vitamins such as niacin, riboflavin, and vitamin C; and minerals including sodium, potassium, and iron. Ginger possesses antifungal, antiviral, and antibacterial properties. Polyphenols, flavones, isoflavones, flavonoids, anthocyanins, coumarins, lignans, catechins, and isocatechins are known to contribute to the health-promoting, anti-inflammatory, anticancer, and antioxidant properties of ginger rhizome [4]. Gingerols and Shogaols are also known to impart anticancer properties to ginger and block the progression of the cell cycle [5,6,7]. Ginger extracts have been reported to be cytotoxic against MDA-MB-231 breast cancer cell lines, HCT 116 colorectal cancer cell lines, colon cancer cell lines, colorectal cell lines, lung cancer cell lines, and cholangiocarcinoma cell lines [7,8,9,10,11,12].

Nanoscience and nanotechnology are presently undergoing significant advancements that will influence diverse industries in due course. Nanotechnology holds significant potential in the field of medicine because of the unique physico-chemical properties of nanomaterials and nanoparticles (NPs). Nanoparticles synthesized using plant extracts offer an eco-friendly, cost-effective, non-toxic, and more biocompatible approach for various applications, including medicine [13]. Nanoparticles exhibit a high surface-to-volume ratio, enhanced permeability and retention, slow and controlled release, high chemical and photo stability, prolonged circulation time, low toxicity, and high specificity [14]. Top–down and the bottom–up approaches are two frequently used methods for synthesizing and creating NPs. The NPs can be obtained through physical, biological, and chemical processes. However, there can be an increased risk of toxicity from the physical and chemical methods. Biologically synthesized NPs offer a more sustainable alternative as it is a more environmentally sustainable, safer, cost-effective, and facile process of producing NPs [15,16]. And perhaps most importantly, biosynthesized NPs can overcome the challenges associated with the chemical and physical methods of NP synthesis.

In that context, nanoformulations derived from ginger have shown enhanced pharmacological, biopharmaceutical, and chemical properties, where the presence of bioactive compounds increased the bioavailability, drug efficacy, drug targeting, and therapeutic value as compared to conventional ginger juice and alcoholic extracts of ginger. Metallic NPs synthesized using plant extracts and metabolites are highly effective and unveil remarkable promise for exploring new NP modifications. Ginger metabolites, such as alkaloids, terpenoids, sugars, polyphenols, flavonoids, phenolics, and proteins, contribute to the bioreduction of metal ions to metallic NPs [2,17]. Metals, such as silver, gold, iron, and selenium, and bulk metal oxides, such as copper oxide, zinc oxide, iron oxide, and magnesium oxide, have been utilized in the manufacture of NPs. Silver NPs synthesized with ginger extracts are reported to have unique beneficial properties like chemical stability, electrical conductivity, and photo-electrochemical, catalytic, antienzymatic, anti-inflammatory, antimicrobial, antiseptic, antioxidant, antiapoptotic, and cytotoxic activity, making them of pharmacological interest, in addition to their being cost-effective [11,13,14,18]. AgNPs produced from plant extracts are reported to remain stable even after prolonged storage [18]. AgNPs have been reported to possess antibacterial efficacy against both multidrug-resistant microorganisms like *Klebsiella pneumoniae*, *E. coli*, *Enterococcus faecalis*, *Streptococcus mutans*, *S. aureus*, and *Candida albicans*, as well as against plant-associated microbes such as *Pseudomonas chlororaphis* [13,19,20,21,22,23,24,25]. Notably, the antioxidant properties of ginger AgNPs and gingerol have been demonstrated by [18,26]. Moreover, AgNPs derived from plant extracts show higher antioxidant activity than either AgNO_3_ or plant extracts tested individually, which indicates the higher reducing capacity of plant-based AgNPs than their free form due to synergistic activities [25]. The strong antioxidant activity of ginger AgNPs due to various phytochemicals helps capture and neutralize free radicals and reactive oxygen and nitrogen species, thereby reducing the risk of other degenerative diseases caused by reactive oxygen species [25,27,28].

**Figure 1:**
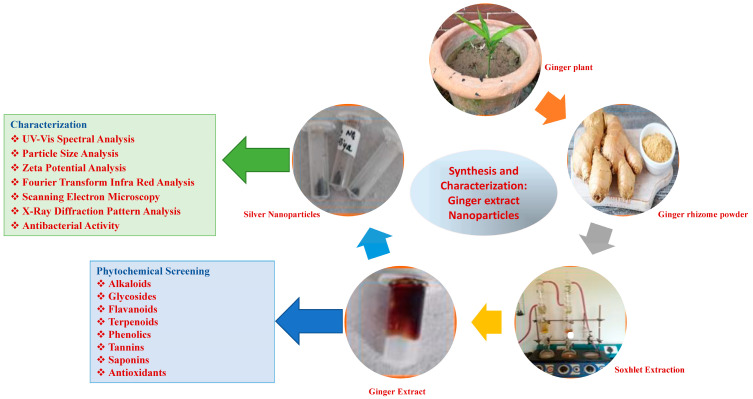
Schematic representation of synthesis and characterization of silver nanoparticles using extracts from ginger rhizome.

This study was aimed at using ginger extract for the synthesis of plant-based AgNPs and the evaluation of the green-synthesized NPs for their antimicrobial activity against pathogens of biomedical interest. To this end, the synthesized NPs were characterized to evaluate their phytochemical, antioxidant, and antibacterial properties to understand their potential application in nanomedicine (Figure 1). Notably, the anticancer activity of the ginger extracts was also studied on Vero cell lines, which is a novelty that has not hitherto been reported.

## 2. Results and Discussion

### 2.1. Phytochemical Analysis and Evaluation of Anticancer Activity of Ginger Extracts

The qualitative phytochemical analysis of a crude extract of ginger revealed the presence of bioactive compounds such as alkaloids, glycosides, flavonoids, phenolics, tannins, saponins, and terpenoids as described in Table 1. The quantitative analysis revealed that the total phenolic content was 1.6 mg/g ± 0.024, the total flavonoid content was 1.8 mg/g ± 0.028, and the total tannin content was 2.5 mg/g ± 0.135. Biomolecules such as phenolics and flavonoids serve as active biocatalysts and as natural stabilizers of metallic NPs [2]. The electron-donating capability and capping properties of the phytochemical compounds like tannins help to stabilize metal NPs [13,29,30,31]. These phytochemicals have been reported to possess antimicrobial properties, as well as serving as antitumor and antioxidant agents [30].

**Figure 2:**
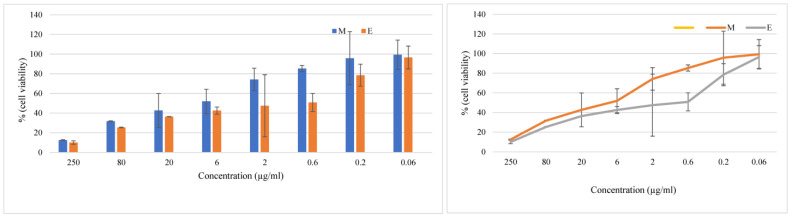
The cytotoxic effects of ginger extracts. Cells were treated with different concentrations of ginger extracts (Methanolic M and Ethanolic E) for 72 h, and cell viability was evaluated by MTT assay. Data are shown as means ± SD (n = 3), compared with the control blank, *p* < 0.05. Fifty percent inhibition was observed at approximately 6 µg/mL for M and 0.6 µg/mL for E.

The in vitro cytotoxicity of plant extracts is commonly the first step of research for anticancer compounds from plant sources as validated by Artun et al. [32]. Vero and HeLa cells have commonly been used for cytotoxic studies of plant compounds [32]; however, such studies have not been reported in ginger. The in vitro cytotoxicity of ginger extracts on Vero cells was, thus, assessed in this study by the MTT assay. The treatment with ginger extract significantly decreased the viability of Vero cells, compared to the control at 72 h (Figure 2). The percentage of cell viability gradually reduced with an increasing concentration, and a 50% growth inhibition was achieved at 0.6 μg/mL concentration for ethanolic extract and 6 μg/mL for methanolic extract. Hence, the IC-50 dose was reflected as 0.6 μg/mL for ethanolic extract and 6 μg/mL for methanolic extract. The cytotoxic properties of ginger can be attributed to the phytoconstituents, including flavanoids, gingerols, shogaols, paradols, and zingerone, which block the cell cycle progression in a dose-dependent manner, as corroborated by Alkhathlan et al. [7] and Jia et al. [6]. The ethanolic extract showed stronger cytotoxicity, which may be due to potent phytochemicals like gingerol and shogaol; these compounds may be more soluble and easier to extract and recover from ethanol as compared to methanol. The extract may be subsequently used in further studies for the preparation of nanoparticles with cytotoxic and anticancer activity. This study confirmed that ginger compounds have potent anticancer activities in vitro and consequently, the compounds responsible for the anticancer activity may also be isolated and purified for preparation of nanoparticles against cancer and can be a potential source for pharmacologically active novel anticancer products.

### 2.2. AgNP Synthesis and Characterization

AgNPs were prepared from AgNO_3_ salt using extracts of the ginger rhizome as reducing and capping agents. Green synthesis of AgNPs from fresh and dried ginger using ethanol and water extracts has been reported as an efficient method by Vijaya et al. [33] and Hu et al. [18]; it does not require any external surfactants and capping or stabilizing agents since this is achieved by the extract itself. The optimal concentrations of the extract and AgNO_3_ (1 mg ginger extract in 1 mL of 1 M AgNO_3_ at 80 °C for 6 h) ensured sufficiently low polydispersity index of the AgNPs and pH 6 yielded the highest quantity of nanoparticles. The formation of NPs was evident by the change in color from pale yellow to dark brown, followed by additional characterization as reported earlier by Garg et al. [34], Ganesan et al. [35], Plaeyao et al. [36], Prasad et al. [37], and Mahardika et al. [13], where the reaction temperature and time, and the concentration of AgNO_3_ are known to influence the shape and size of the ensuing NPs.

**Figure 3:**
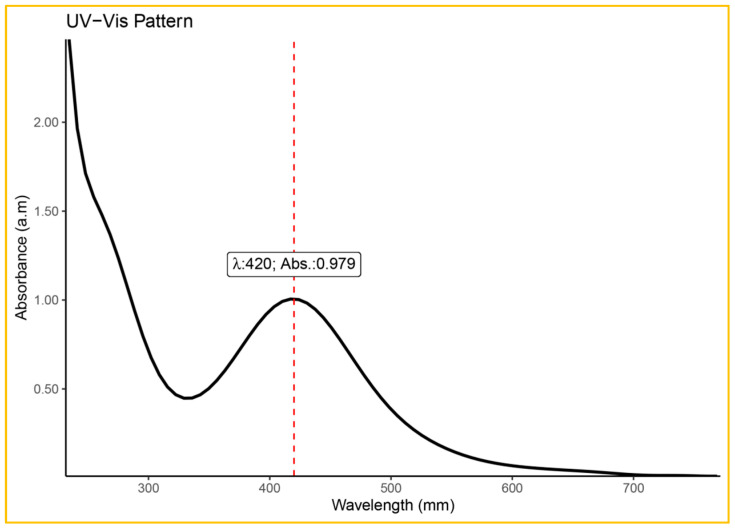
UV–visible absorbance spectra of AgNPs of ginger rhizome extract.

UV-Vis spectral analysis confirmed the successful synthesis of NPs in the range of 400–500 nm. UV–visible absorption spectra showed a single, prominent broad surface plasmon resonance peak at 420 nm indicating the synthesis of AgNPs (Figure 3). This feature could be attributed to the presence of spherical NPs as indicated in previous studies [18,35]. Yadav et al. [38] reported that the plasmon resonance band at 420 nm supported the reduction of AgNO_3_ to AgNP.

**Figure 4:**
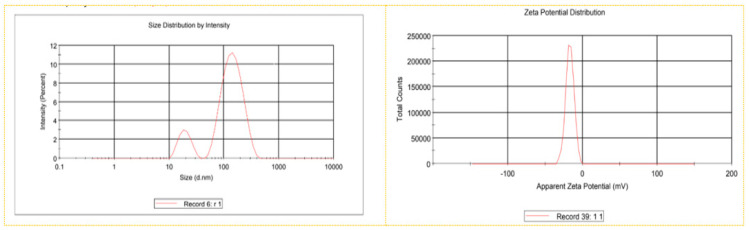
Particle size distribution and zeta potential curve of AgNPs synthesized using ginger rhizome extract.

The polydispersity index (PDI) measures the uniformity of NPs, indicating the size distribution of the NPs with a value that ranges from 0 to 1. As shown in Figure 4 and Table 2 for the ginger-derived AgNPs, the particle size distribution curve reveals that the NPs were polydispersed, with an average diameter of 81.6 nm and a PDI of 0.510. The zeta potential analysis to determine the surface charge showed an average value of −17.1 ± 5.2 mV and a conductivity of 0.033 mS/cm. Ostensibly, the presence of functional groups from the ginger extract imparted a relatively high negative charge value on the NP surface. This charge would have been responsible for the high dispersity, colloidal nature, and long-term stability of AgNPs because of a negative–negative repulsion as discussed [39].

**Figure 5:**
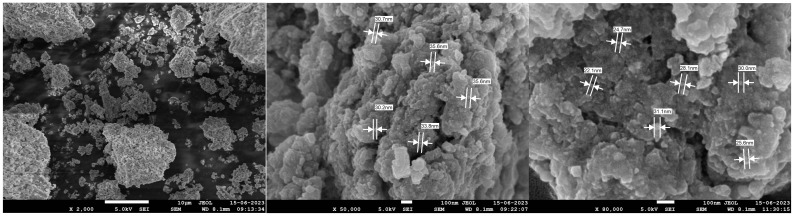
SEM image of AgNPs synthesized using ginger rhizome extract.

SEM analysis confirmed the spherical nature of the particles within the diameter range 30–120 nm and an average size of 80–100 nm. The NPs were well distributed with aggregation also noted (Figure 5). These size characteristics agree with those previously reported for AgNPs with high biological activity [25,29]. Indeed, AgNPs tend to agglomerate due to their high surface energy and surface tension [38].

**Figure 6:**
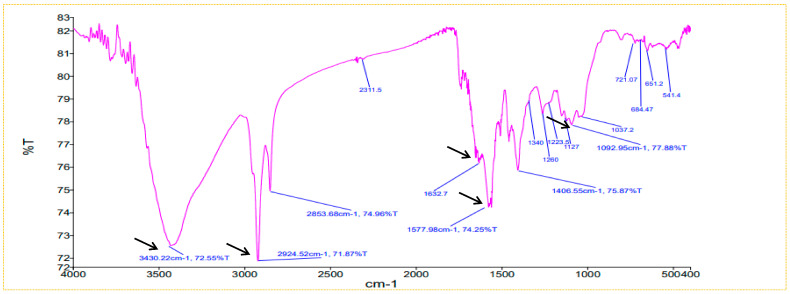
FTIR spectra of AgNPs synthesized using ginger rhizome extract.

The chemical nature and the presence of phytochemicals and functional groups on NP surfaces can be determined by FTIR analysis. In this study, the FTIR analysis revealed the association of various functional groups of biomolecules including ethers, esters, alcohols, and carboxylic acids with the surface of the NPs. These functional groups ostensibly helped in the reduction and stabilization of particles. The O-H stretching of alcohols and phenols at 3430 cm^−1^, O-H stretching of carboxylic acids at 2924 cm^−1^, N-H bend primary amines at 1632 cm^−1^, alkanes at 1645 cm^−1^, N-O asymmetric stretch at 1577 cm^−1^, N-H stretch vibration in amide links of the proteins at 1639 cm^−1^, and C-O stretching of alcohol, ether, ester, and carboxylic acids at 1127 and 1092 cm^−1^ were observed (Figure 6). These associations agreed with previously reported data by Alagesan and Venugopal [29]. The majority of the peaks indicated the presence of phenolics, steroids, tannins, flavonoids, terpenoids, alkaloids, and saponins, as previously reported by Ramzan et al. [22]. The FTIR analysis displayed the involvement of different functional groups as capping and stabilizing agents in the stabilization of NPs.

**Figure 7:**
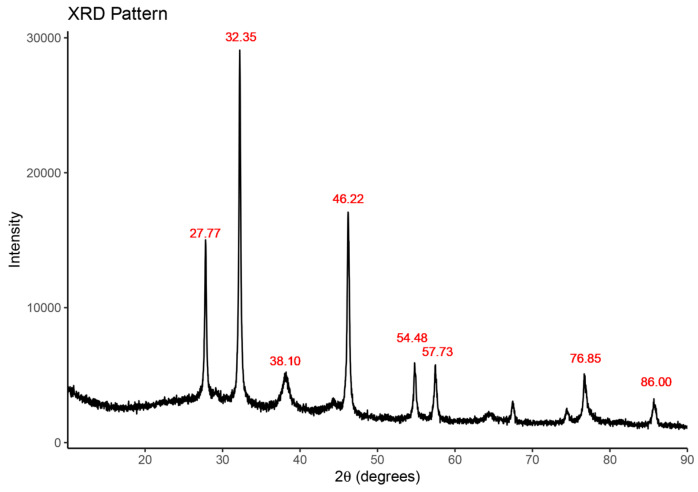
XRD patterns of AgNPs synthesized using ginger rhizome extract.

XRD analysis of the biosynthesized NPs showed the strong peaks at 2ᴓ = 28, 32, 46. When compared with the standard powder diffraction card of JCPDS, silver file No. 04-0783, these distinct clear peaks confirmed the high purity and crystalline nature of the NPs [40,41]. XRD analysis further revealed diffracted intensities from 20° to 90° (Figure 7). The strong Bragg reflections at approximately 28, 32, and 46 could be indexed according to the facets of face-centered cubic crystal structure of silver [42]. The additional peaks have been identified to be due to AgNO_3_, which might have not been reduced and hence remained in the sample in minute quantity. The phytochemicals present in the ginger extract, including flavonoids, alkaloids, tannins, and saponins, are reported to act as capping agents and impart the average crystallite size of metallic NPs [43].

### 2.3. Analysis of Antioxidant and Antibacterial Activities of AgNPs

The antioxidant activity of the AgNPs synthesized with the ethanolic extracts of ginger was estimated by the percentage inhibition of DPPH radicals. The particles showed high activity in scavenging DPPH free radicals. The ginger rhizome extract and AgNPs exhibited high antioxidant activity of 75.4% and 84.2%, respectively, which is due to the presence of hydroxyl groups and solubilizing side chains of polyphenolic compounds like gingerols, shogaols, paradols, and gingerdions, as corroborated by Ali et al. [41] and Hu et al. [18]. Gingerols and shogaols in ginger are known to effectively scavenge DPPH [44]. Aqueous and ethanol extracts of ginger have various natural antioxidants, with significant scavenging ability against superoxide radicals and lipid peroxidation. The aqueous extract contains predominantly shogaol, while ethanol extract contains various gingerols and shogaols like those imparting superior antioxidant properties as corroborated by Ongtanasup et al. [45]. The ethanol extract of dry ginger has been reported to exhibit good antioxidant properties, and it can be used as a natural antioxidant [18]. The antioxidant activity of ginger has been demonstrated to be unaffected by thermal denaturation [30].

The prevalence of multidrug-resistant microorganisms has increased, leading to a rise in infectious diseases and increased mortality rates worldwide. Silver nanoformulations, both chemically synthesized and green synthesized, are known to inhibit the growth of microorganisms such as bacteria by affecting bacterial membranes [46]. Therefore, the ethanolic extract and AgNPs obtained from ginger rhizomes were tested for antibacterial activity against the Gram-positive bacterium *Bacillus subtilis* and the Gram-negative bacteria *Escherichia coli*, *Pseudomonas aeruginosa*, and *Staphylococcus aureus*. To this end, the antibiotic streptomycin was used as the positive control, while DMSO was used as the negative control. Table 3 shows the zone of inhibition of the four bacteria, indicating that the NPs were effective against all the strains.

It follows that there is an enhanced inhibitory effect of AgNPs due to the ginger extract. The bactericidal activity is linked to the active biomolecules like gingerol, zingerone, and shogaol present in ginger. The proteins, tannins, saponins terpenoids, and flavonoids present in the extract also interfere with the bacterial cell membrane [24]. The antimicrobial activity of AgNPs is related to the small particle size and high surface area and surface-to-volume ratio of NPs, which result in an increased contact area with microorganisms and thus an increase in antimicrobial activity [14,44]. The antibacterial activity of green synthesized ginger nanoparticles has also been studied earlier [25,33,47]. The enhanced antibacterial activity of AgNPs is due to the disruption of the bacterial membrane which was because of the synergistic effect of silver and plant antioxidants, and which can be exploited against multidrug-resistant bacteria as reported by Ganesan et al. [35]. Taken together, it can be concluded that AgNPs are capable of imparting high antibacterial efficacy and can offer an advantage in broad-spectrum antibacterial and anticancer drug preparation, and also in nanomedicine, as corroborated by Plaeyao et al. [36] and Swapna et al. [48].

## 3. Materials and Methods

### 3.1. Preparation of Plant Extract

Fresh rhizomes of ginger (*Zingiber officinale* (L.) Rosc.) were acquired from a local market. Rhizomes were washed to remove debris, sliced, and spread onto drying glass trays in a drying hot air oven (NSW-151) at 60 °C. The dried samples were ground with a mortar and pestle before extraction. The powder (10 g) was extracted with 100 mL of distilled water, 95% ethanol, and 80% methanol separately for eight hours in a Soxhlet apparatus at 80 °C. The extracts were filtered by Whatman No. 1 filter paper and poured into glass petri plates concentrated by evaporation in hot-air oven at 45 °C. The extract was removed from the petri plate and stored at 4 °C for further analysis.

### 3.2. Green Synthesis and Characterization of Silver Nanoparticles

AgNP synthesis was optimized by screening different concentrations of AgNO_3_ and ethanolic extract of ginger, at a range of pH levels 6–8, temperature 40–80 °C, and duration. A total of 50 mg of Ginger extract was slowly added to 50 mL of 1 M Silver nitrate under magnetic stirring and pH was controlled by dropwise addition of NaOH solution, and subsequent incubation at 80 °C for 6 h. A color change from pale yellow to brown indicated the formation of AgNPs.

The synthesized AgNPs were obtained after centrifugation at 8000 rpm for 15 min at 37 °C and washing with ethanol. The particles were dried and stored at 4 °C until use. The absorbance of the NPs was analyzed using a UV–Vis spectrophotometer (Shimadzu UV-2450, Milton Keynes, UK) at 200–800 nm. The size and zeta potential of the NPs and the particle size distribution were measured by dynamic light scattering (Nano-ZS90 Malvern Instruments Ltd., Malvern, UK). FTIR (Fourier Transform Infrared Spectroscopy) measurements were recorded under vacuum optics on a Perkin-Elmer Spectrum IR (Version 10.6.2) with a resolution of 4 cm^−1^ in the spectral region of 4000 cm^−1^ to 400 cm^−1^ to analyze the functional groups associated with biomolecules on the particle surface. Particle morphology was studied using Field Emission Scanning Electron Microscope SEM (JSM-7800F, Jeol, Tokyo, Japan). The X-ray diffraction (XRD) properties of the synthesized NPs were evaluated on a multifunctional X-ray diffractometer (Rigaku, Tokyo, Japan) using Cu-Kα radiation of wavelength λ = 1.541 Å of green synthesized Ag NPs. The diffractogram was compared with the standard powder diffraction card of JCPDS, silver file No. 04-0783. The crystalline size of the prepared nanoparticles was determined by using Scherrer’s equation as follows: D ≈ 0.9λ/βcosθ, where D is the crystal size, λ is the wavelength of X-ray, is the Bragg angle in radians, and B is the full width at half maximum of the peak in radians.

### 3.3. Phytochemical Analysis

The qualitative and quantitative phytochemical analyses of ginger extracts (distilled water, ethanolic, methanolic) were carried out according to Alagesan and Venugopal [29] and Kathirvel and Sujatha [49]. Specifically, the content of alkaloids (Wagner’s test), cardiac glycosides (Keller Killiani test), flavanoids (Lead Acetate test), terpenoids (Salkowski test), phenolics (Ferric Chloride test), tannins (Braymer’s test), and saponins (Foam/Froth test) was determined by these assays. The content of total phenol, flavonoids, and tannins was determined by a spectrophotometric assay/Folin–Ciocalteau phenol method and was expressed as micrograms of gallic acid/catechin/tannic acid equivalents, respectively, per milligram of extract.

### 3.4. Evaluation of the Antimicrobial and Antioxidant Activity

The antimicrobial activity of ginger extract and ginger-synthesized AgNPs was assessed at a concentration of 10 mg/mL against 1 mL culture of *Escherichia coli*, *Bacillus subtilis*, *Pseudomonas aeruginosa*, and *Staphylococcus aureus* in Muller Hilton agar plate at pH 7.4 by the Kirby Bauer disc diffusion method using the antibiotic Streptomycin (10 µg/disc) as a positive control, while dimethyl sulfoxide served as the negative control. The zone of inhibition around the wells was measured in mm after the 24 h incubation period at 37 °C. The antioxidant activities of crude extracts and the AgNPs were determined by the DPPH radical scavenging method according to Nagajyothi et al. [50] and Mensor et al. [50] using 5 mL of DPPH (0.1 M) and 5 mL of AgNPs (50 µg/mL) incubated in dark at room temperature for 30 min. The reduction of DPPH radical was measured as absorbance at 517 nm by UV–Vis spectrophotometer. DPPH scavenging ability was calculated as

$$\mathrm{DPPH}\;\mathrm{RSA}\;\%=\frac{Abs\;of\;Control/DPPH-Abs\;of\;Sample}{Abs\;of\;Control}$$

The overall experimental scheme followed in the study is illustrated in Figure 1.

### 3.5. Evaluation of the Anticancer Activity on Cell Lines by Cytotoxicity Assay

Cytotoxicity assays on the African green monkey kidney epithelial (Vero) cell lines were performed according to Kumar et al. 2020. Vero cell line was obtained from the National Centre for Veterinary Type Cultures (NCVTV), Hisar, and was maintained in Dulbecco’s Modified Eagle Medium (DMEM) (Sigma-Aldrich, St. Louis, MO, USA) supplemented with 10% fetal bovine serum (FBS), 10,000 units penicillin, 10 mg streptomycin/mL (Sigma-Aldrich, St. Louis, MO, USA), and 3.7 g/L sodium bicarbonate at pH 7.4. Cells were maintained at 37 °C under a humidified 5% CO_2_ atmosphere in CO_2_ incubator (New Brunswick™, Galaxy^®^ 170 R, Eppendorf AG, Hamburg, Germany)

A confluent cell monolayer of Vero cells with the cell density of 4 × 10^4^ cells/well were seeded in 96-well plates and incubated at 37 °C in the presence of 5% CO_2_ for 24 h. After incubation, the Vero cells were treated with different concentrations of plant extract (250, 80, 20, 6, 2, 0.6, 0.2, 0.06 μg/mL) in triplicate wells using DMSO as negative control and incubated at 37 °C in a 5% CO_2_ incubator for 72 h. The cell viability was evaluated using the MTT colorimetric assay. MTT solution (5 mg/mL in phosphate buffered saline) was pipetted into each well followed by a 4 h incubation period at 37 °C in the 5% CO_2_ incubator. The formazan crystals were dissolved in 100 μL DMSO and the absorbance was determined at 570 nm. The percentage of cytotoxicity was calculated with reference to the negative control cells. The concentration depicting 50% reduction in cell viability was considered as IC50 (50% inhibitory concentration).

### 3.6. Statistical Analysis

Statistical analysis was carried out using the SPPS 13.0 using a significance level of *p* < 0.05. Analysis of variance (ANOVA) in a completely randomized design and Tukey’s multiple-range post hoc tests were used to determine significant differences between samples. All measurements were performed in triplicate and averaged. Values were expressed as mean ± standard deviation.

## 4. Conclusions

This work explored the array of phytochemical, antioxidant, anticancer, and antibacterial potential of ginger rhizome by using its extract for the rapid, simple, and reproducible phyto-fabrication of silver nanoparticles and evaluating the same for antimicrobial efficacy against critically important pathogens. Our results indicated that ginger extract or its constituents separately may have clinical effects for therapeutic interventions. The comprehensive findings highlight the biomedical advantages of ginger and its derived nanoparticles, and emphasize their potential incorporation into drugs at very low concentrations. The findings from the study show that ginger can be exploited as a future phytomedicine, since it possesses antioxidant, antibiotic, antiemetic, antidiabetic, antiangiogenic, cardiovascular, anticoagulant, anti-inflammatory, antiemetic, antinociceptive, antimicrobial, antiobesity, antidiabetic, antitussive, immunomodulatory, cytotoxic, and chemo-preventive properties owing to its bio-active constituents. Ginger and its phytochemical constituents, such as gingerols, shogaols, paradols, dihydroparadols, diarylheptanoids, zingiberene, and phellandrene, are responsible for its therapeutic and pharmacological properties. These bioactive phytochemicals can be combined with the metal ions to form metallic nanoparticles, which exhibit improved bioactivity, decreased toxicity, and enhanced physical stability. As proposed for agriculture by Vaidya et al. [5], advancing knowledge in green chemistry involving biogenic synthesis of nanoscale bio-actives using precursors from plants such as ginger will facilitate investigations on constituent secondary metabolites, molecular mechanisms, and signaling pathways, and the defense-related gene responses can assist in manufacturing metallic NPs with plant extracts for antimicrobial and anticancer applications in healthcare industries. Green synthesized sustainable and non-toxic NPs with eminent medicinal properties will benefit the pharmaceutical industry by providing a platform to produce biocompatible and bioactive agents in a sustainable fashion.

## Figures and Tables

**Table 1:** Phytochemical screening/qualitative analysis of ginger rhizome extract (+ = detected; ++ = strongly detected; - = not detected).

Phytochemicals	Test	Distilled Water	Ethanolic Extract	Methanolic Extract
Alkaloids	Wagner’s test	++	++	++
Glycosides	Keller Killiani test	+	+	+
Flavanoids	Lead Acetate test	+	++	+
Terpenoids	Salkowski test	+	++	+
Phenolics	Ferric Chloride test	+	+	+
Tannins	Braymer’s test	-	++	++
Saponins	Foam/Froth test	+	++	++

**Table 2:** Particle size distribution and zeta potential analysis of AgNPs synthesized using ginger rhizome extract.

Characterisation	Value
Z-average, d. nm	81.6
Polydispersity index	0.510
Zeta potential (mV) at pH 7	−17.1 ± 5.52 (STD DEV)
Conductivity (mS/cm)	0.0330

**Table 3:** Antibacterial activities of ginger rhizome extract and ginger-derived AgNPs together with their corresponding zones of inhibition (mm).

Bacterial Strain	Zone of Inhibition (mm)		
	Antibiotic Streptomycin	Ethanolic Extract	Methanolic Extract
*1. Escherichia coli* (G−)	18	4	5
*2. Bacillus subtilis* (G+)	11	3	5
*3. Pseudomonas aeruginosa* (G−)	9	10	9
*4. Staphylococcus aureus* (G+)	10	4	4
**Bacterial Strain**	**Zone of Inhibition (mm)**		
	**DMSO**	**Silver Nanoparticles**	**Antibiotic Streptomycin**
*1. Escherichia coli* (G−)	Negative	5	5
*2. Bacillus subtilis* (G+)	Negative	4	5
*3. Pseudomonas aeruginosa* (G−)	Negative	8	6
*4. Staphylococcus aureus* (G+)	Negative	4	8

## Data Availability

All data are contained within the article.

## References

[1] Sharma S., Shukla M.K., Sharma K.C., Tirath, Kumar L., Anal J.M.H., Upadhyay S.K., Bhattacharyya S., Kumar D. (2023). Revisiting the therapeutic potential of gingerols against different pharmacological activities. Naunyn-Schmiedeberg’s Arch. Pharmacol..

[2] Hazim I., Abd K.Y., Abachi F.T. (2020). Newly formulated extract of *Zingiber officinale* as reducing agent for Silver nitrate Nanoparticals. Pharma Innov. J..

[3] Ghasemzadeh A., Jaafar H.Z., Baghdadi A., Tayebi-Meigooni A. (2018). Formation of 6-, 8-and 10-shogaol in ginger through application of different drying methods: Altered antioxidant and antimicrobial activity. Molecules.

[4] Mukjerjee S., Karati D. (2022). A mechanistic view on phytochemistry, pharmacognostic properties, and pharmacological activities of phytocompounds present in *Zingiber officinale*: A comprehensive review. Pharmacol. Res.-Mod. Chin. Med..

[5] Shanmugam K.R., Shanmugam B., Venkatasubbaiah G., Ravi S., Reddy K.S., Chakraborti S. (2022). Recent Updates on the Bioactive Compounds of Ginger (*Zingiber officinale*) on Cancer: A Study with Special Emphasis of Gingerol and Its Anticancer Potential. Handbook of Oxidative Stress in Cancer: Therapeutic Aspects.

[6] Jia Y., Li X., Meng X., Lei J., Xia Y., Yu L. (2023). Anticancer perspective of 6-shogaol: Anticancer properties, mechanism of action, synergism and delivery system. Chin. Med..

[7] Alkhathlan A.H., Al-Abdulkarim H.A., Khan M., Khan M., Alkholief M., Alshamsan A., Almomen A., Albekairi N., Alkhathlan H.Z., Siddiqui M.R.H. (2021). Evaluation of the Anticancer Activity of Phytomolecules Conjugated Gold Nanoparticles Synthesized by Aqueous Extracts of *Zingiber officinale* (Ginger) and *Nigella sativa* L. Seeds (Black Cumin). Materials.

[8] Plengsuriyakarn T., Viyanant V., Eursitthichai V., Tesana S., Chaijaroenkul W., Itharat A., Na-Bangchang K. (2012). Cytotoxicity, toxicity, and anticancer activity of *Zingiber officinale* Roscoe against cholangiocarcinoma. Asian Pac. J. Cancer Prev..

[9] Malmir S., Ebrahimi A., Mahjoubi F. (2020). Effect of ginger extracts on colorectal cancer HCT-116 cell line in the expression of MMP-2 and KRAS. Gene Rep..

[10] Khdary N.H., Alangari A., Katubi K.M., Alanazi M., Alhassan A.A., Alzahrani S.D., Khan Z., Alanazi I.O. (2023). Synthesis of Gingerol-Metals Complex and In-Vitro Cytotoxic Activity on Human Colon Cancer Cell Line. Cancer Manag. Res..

[11] Osman A., Eldin I.M., Elhag A.M., Elhassan M.M., Ahmed E.M. (2020). In-vitro Anticancer and Cytotoxic Activity of Ginger Extract on Human Breast Cell Lines. Khartoum J. Pharm. Sci..

[12] Nachvak S.M., Soleimani D., Rahimi M., Azizi A., Moradinazar M., Rouhani M.H., Halashi B., Abbasi A., Miryan M. (2022). Ginger as an anticolorectal cancer spice: A systematic review of in vitro to clinical evidence. Food Sci. Nutr..

[13] Mahardika D.P., Utomo F., Desdicha V., Asrul Z. (2021). Antibacterial activity of phytogenic silver nanoparticles using domestic herbs plant extract. J. Phys. Conf. Ser..

[14] Kebede Urge S., Tiruneh Dibaba S., Belay Gemta A. (2023). Green synthesis method of ZnO nanoparticles using extracts of *Zingiber officinale* and garlic bulb (*Allium sativum*) and their synergetic effect for antibacterial activities. J. Nanomater..

[15] Karmous I., Taheur F.B., Zuverza-Mena N., Jebahi S., Vaidya S., Tlahig S., Mhadhbi M., Gorai M., Raouafi A., Debara M. (2022). Phytosynthesis of zinc oxide nanoparticles using *Ceratonia siliqua* L. and evidence of antimicrobial activity. Plants.

[16] Karmous I., Vaidya S., Dimkpa C., Zuverza-Mena N., da Silva W., Barroso K.A., Milagres J., Bharadwaj A., Abdelraheem W., White J.C. (2023). Biologically synthesized zinc and copper oxide nanoparticles using *Cannabis sativa* L. enhance soybean (*Glycine max*) defense against *Fusarium virguliforme*. Pestic. Biochem. Physiol..

[17] Kamal A., Zaki S., Shokry H., Abd-El-Haleem D. (2020). Using ginger extract for synthesis of metallic nanoparticles and their applications in water treatment. J. Pure Appl. Microbiol..

[18] Hu D., Gao T., Kong X., Ma N., Fu J., Meng L., Duan X., Hu C.Y., Chen W., Feng Z. (2022). Ginger (*Zingiber officinale*) extract mediated green synthesis of silver nanoparticles and evaluation of their antioxidant activity and potential catalytic reduction activities with Direct Blue 15 or Direct Orange 26. PLoS ONE.

[19] Dimkpa C.O., Calder A., Gajjar P., Merugu S., Huang W., Britt D.W., McLean J.E., Johnson W.P., Anderson A.J. (2011). Interaction of silver nanoparticles with an environmentally beneficial bacterium, *Pseudomonas chlororaphis*. J. Hazard. Mater..

[20] Hiremath J., Rathod V., Ninganagouda S., Singh D., Prema K. (2014). Antibacterial activity of Silver Nanoparticles from *Rhizopus* spp. against Gram negative *E. coli* MDR strains. J. Pure Appl. Microbiol..

[21] Tariq F., Ahmed N., Afzal M., Khan M.A.U., Zeshan B. (2020). Synthesis, Characterization and antimicrobial activity of *Bacillus subtilis*-derived silver nanoparticles against multidrug-resistant bacteria. Jundishapur J. Microbiol..

[22] Ramzan M., Karobari M.I., Heboyan A., Mohamed R.N., Mustafa M., Basheer S.N., Desai V., Batool S., Ahmed N., Zeshan B. (2022). Synthesis of silver nanoparticles from extracts of wild ginger (*Zingiber zerumbet*) with antibacterial activity against selective multidrug resistant oral bacteria. Molecules.

[23] Yadi M., Azizi M., Dianat-Moghadam H., Akbarzadeh A., Abyadeh M., Milani M. (2022). Antibacterial activity of green gold and silver nanoparticles using ginger root extract. Bioprocess Biosyst. Eng..

[24] Reda M., Ashames A., Edis Z., Bloukh S., Bhandare R., Abu Sara H. (2019). Green synthesis of potent antimicrobial silver nanoparticles using different plant extracts and their mixtures. Processes.

[25] Abdellatif A.A., Alhathloul S.S., Aljohani A.S., Maswadeh H., Abdallah E.M., Hamid Musa K., El Hamd M.A. (2022). Green synthesis of silver nanoparticles incorporated aromatherapies utilized for their antioxidant and antimicrobial activities against some clinical bacterial isolates. Bioinorg. Chem. Appl..

[26] Roja B. (2019). Green Synthesis of Silver Nanoparticles Using Ginger Extract and Its Antioxidant (In Vitro) and Anticancer (Insilico) Study. Sch. Natl. Sch. Leadersh..

[27] Wang Y., Chinnathambi A., Nasif O., Alharbi S.A. (2021). Green synthesis and chemical characterization of a novel anti-human pancreatic cancer supplement by silver nanoparticles containing *Zingiber officinale* leaf aqueous extract. Arab. J. Chem..

[28] Jing Y., Cheng W., Ma Y., Zhang Y., Li M., Zheng Y., Zhang D., Wu L. (2022). Structural characterization, antioxidant and antibacterial activities of a novel polysaccharide from *Zingiber officinale* and its application in synthesis of silver nanoparticles. Front. Nutr..

[29] Alagesan V., Venugopal S. (2019). Green synthesis of selenium nanoparticle using leaves extract of withania somnifera and its biological applications and photocatalytic activities. Bionanoscience.

[30] Shalaby T.I., Mahmoud O.A., El Batouti G.A., Ibrahim E.E. (2015). Green synthesis of silver nanoparticles: Synthesis, characterization and antibacterial activity. Nanosci. Nanotechnol..

[31] Muchtaromah B., Wahyudi D., Ahmad M., Ansori A.N.M., Annisa R., Hanifah L. (2021). Chitosan-tripolyphosphate nanoparticles of mango ginger (*Curcuma mangga*) extract: Phytochemical screening, formulation, characterization, and antioxidant activity. Pharmacogn. J..

[32] Tugba Artun F., Karagoz A., Ozcan G., Melikoglu G., Anil S., Kultur S., Sutlupinar N. (2016). In vitro anticancer and cytotoxic activities of some plant extracts on HeLa and Vero cell lines. J. BU ON.

[33] Vijaya J.J., Jayaprakash N., Kombaiah K., Kaviyarasu K., Kennedy L.J., Ramalingam R.J., Al-Lohedan H.A., Mansoor-Ali V., Maaza M. (2017). Bioreduction potentials of dried root of *Zingiber officinale* for a simple green synthesis of silver nanoparticles: Antibacterial studies. J. Photochem. Photobiol. B Biol..

[34] Garg A., Pandey P., Sharma P., Shukla A. (2016). Synthesis and characterization of silver nanoparticle of ginger rhizome (*Zingiber officinale*) extract: Synthesis, characterization and anti diabetic activity in streptozotocin induced diabetic rats. Eur. J. Biomed. Pharm. Sci..

[35] Ganesan S., Palanichamy P., Selvam G. (2021). Biosynthesis and characterization of silver nanoparticles using ginger spent and their antibacterial activity. Curr. Bot..

[36] Plaeyao K., Kampangta R., Korkokklang Y., Talodthaisong C., Saenchoopa A., Thammawithan S., Latpala K., Patramanon R., Kayunkid N., Kulchat S. (2023). Gingerol extract-stabilized silver nanoparticles and their applications: Colorimetric and machine learning-based sensing of Hg (ii) and antibacterial properties. RSC Adv..

[37] Prasad R.D., Prasad S.R., Shrivastav O.P., Kanthe A., Waghmare S., Shaikh V.S., Doke K.M., Prasad N.R., Nazeruddin G.M., Shaikh Y.I. (2023). Biogenic Synthesis of Nano-Silver and Their Anti-Microbial Efficacy. ES Food Agrofor..

[38] Yadav S.R., Hodiwala A., Patil P., Thakur M. (2021). Physiochemical characterization & antibacterial properties of biologically synthesized silver nanoparticles from aqueous extracts of ginger. J. Med. Pharm. Allied Sci..

[39] Mukherjee S., Chowdhury D., Kotcherlakota R., Patra S., Vinothkumar B., Bhadra M.P., Sreedhar B., Patra C.R. (2014). Potential theranostics application of bio-synthesized silver nanoparticles (4-in-1 system). Theranostics.

[40] Lanje A.S., Sharma S.J., Pode R.B. (2010). Synthesis of silver nanoparticles: A safer alternative to conventional antimicrobial and antibacterial agents. J. Chem. Pharm. Res..

[41] Ali A.M.A., El-Nour M.E.M., Yagi S.M. (2018). Total phenolic and flavonoid contents and antioxidant activity of ginger (*Zingiber officinale* Rosc.) rhizome, callus and callus treated with some elicitors. J. Genet. Eng. Biotechnol..

[42] Riaz M., Sharafat U., Zahid N., Ismail M., Park J., Ahmad B., Rashid N., Fahim M., Imran M., Tabassum A. (2022). Synthesis of biogenic silver nanocatalyst and their antibacterial and organic pollutants reduction ability. ACS Omega.

[43] Haider A., Ijaz M., Ali S., Haider J., Imran M., Majeed H., Shahzadi I., Ali M.M., Khan J.A., Ikram M. (2020). Green synthesized phytochemically (*Zingiber officinale* and *Allium sativum*) reduced nickel oxide nanoparticles confirmed bactericidal and catalytic potential. Nanoscale Res. Lett..

[44] Rigane G., Mnif S., Ben Salem R. (2018). One step purification of 6-shogaol from *Zingiber officinale* Rosco, a phenolic compound having a high effectiveness against bacterial strains. Rev. Roum. Chim..

[45] Ongtanasup T., Kamdenlek P., Manaspon C., Eawsakul K. (2024). Green-synthesized silver nanoparticles from *Zingiber officinale* extract: Antioxidant potential, biocompatibility, anti-LOX properties, and in silico analysis. BMC Complement. Med. Ther..

[46] Almatroudi A. (2020). Silver nanoparticles: Synthesis, characterisation and biomedical applications. Open Life Sci..

[47] Vijayakumar G., Kesavan H., Kannan A., Arulanandam D., Kim J.H., Kim K.J., Song H.J., Kim H.J., Rangarajulu S.K. (2021). Phytosynthesis of copper nanoparticles using extracts of spices and their antibacterial properties. Processes.

[48] Swapna B., Chandrasekhar K., Madhuri D., Sumathi G. (2022). Biological Synthesis and Characterization of Silver Nanoparticles of Camelia Sinensis and Evaluation of Its Biological Activity. NeuroQuantology.

[49] Kathirvel A., Sujatha V. (2016). Phytochemical studies, antioxidant activities and identification of active compounds using GC–MS of *Dryopteris cochleata* leaves. Arab. J. Chem..

[50] Nagajyothi P.C., Cha S.J., Yang I.J., Sreekanth T., Kim K.J., Shin H.M. (2015). Antioxidant and anti-inflammatory activities of zinc oxide nanoparticles synthesized using *Polygala tenuifolia* root extract. J. Photochem. Photobiol. B Biol..

